# Age-related changes in the temporal dynamics of executive control: a study in 5- and 6-year-old children

**DOI:** 10.3389/fpsyg.2014.00831

**Published:** 2014-07-29

**Authors:** Joanna Lucenet, Agnès Blaye

**Affiliations:** CNRS, Laboratoire de Psychologie Cognitive, UMR 7290, Aix-Marseille UniversitéMarseille, France

**Keywords:** goal setting, reactive control, proactive control, context processing, cognitive development, executive functions

## Abstract

Based on the Dual Mechanisms of Control theory (Braver et al., 2007), this study conducted in 5- and 6-year-olds, tested for a possible shift between two modes of control, proactive vs. reactive, which differ in the way goal information is retrieved and maintained in working memory. To this end, we developed a children-adapted version of the AX-Continuous-Performance Task (AX-CPT). Twenty-nine 5-year-olds and 28-6-year-olds performed the task in both low and high working-memory load conditions (corresponding, respectively, to a short and a long cue-probe delay). Analyses suggested that a qualitative change in the mode of control occurs within the 5-year-old group. However, quantitative, more graded changes were also observed both within the 5-year-olds, and between 5 and 6 years of age. These graded changes demonstrated an increasing efficiency in proactive control with age. The increase in working memory load did not impact the type of dynamics of control, but had a detrimental effect on sensitivity to cue information. These findings highlight that the development of the temporal dynamics of control can be characterized by a shift from reactive to proactive control together with a more protracted and gradual improvement in the efficiency of proactive control. Moreover, the question of whether the observed shift in the mode of control is task dependant is debated.

## INTRODUCTION

Executive control, defined as the ability to regulate, coordinate, and guide one’s thoughts and behaviors toward goals, is probably one of the most critical aspects of human cognition. Indeed, executive control is involved in the development of many cognitive and social skills during childhood such as language (Deak, 2003), theory of mind (Carlson and Moses, 2001; for a review see Miller and Marcovitch, 2012), reading, reasoning and arithmetic (Blair and Razza, 2007; Clark et al., 2013), and emotion regulation (Carlson and Wang, 2007; Eisenberg and Sulik, 2012). It is now well accepted that executive control dramatically develops between the ages of 3 and 6 years (Wright et al., 2003; Carlson et al., 2004; Chevalier et al., 2012). Although these developmental changes have been viewed as resulting merely from an increase in the efficiency of control, recent work suggests that age-related qualitative differences in the control strategies used may also contribute to this development (Chatham et al., 2009; Dauvier et al., 2012; Chevalier et al., 2013). The aim of the present study was to assess whether qualitative changes in the mode of control might occur between the ages of 5 and 6. Specifically, we investigated potential age-related differences in the use of two modes of control proactive vs. reactive which differ in terms of the activation and maintenance of goal representations. To this end, 5- and 6-year-old children were presented with an adapted version of the AX-Continuous Performance Task (Braver et al., 2001).

Executive control is traditionally viewed as composed of three functions: inhibition, flexibility, and working memory updating (Miyake et al., 2000; Lehto et al., 2003; Carlson, 2005). Despite their partial independence, there is converging evidence that these functions share a common base (Miyake et al., 2000; Friedman et al., 2008; Miyake and Friedman, 2012). These authors have proposed that active maintenance of a goal representation and its use to bias task processing under conditions of interference could account for this common core component. Recent empirical work supports this hypothesis and suggests that the activation and maintenance of task–goal information may play a critical role in efficient control, both in adults (Baddeley et al., 2001; Rubinstein et al., 2001; Emerson and Miyake, 2003; Gruber and Goschke, 2004) and in children (Morton and Munakata, 2002; Zelazo et al., 2003; Towse et al., 2007; Chevalier and Blaye, 2009; Chevalier et al., 2010; Blaye and Chevalier, 2011). Developmental studies reveal that the representation and active maintenance of task–goal information improve from childhood to adulthood (Karbach and Kray, 2007; Chevalier and Blaye, 2009; Chevalier et al., 2010, 2012).

Preschool-aged children’s poor flexibility has recently been shown to depend, at least in part, on failures of goal maintenance (Marcovitch et al., 2007, 2010) and in goal representation (Chevalier and Blaye, 2009). Marcovitch et al. (2007, 2010) used a variant of the Dimensional Change Card Sorting task (DCCS; Zelazo et al., 1996). The DCCS task consists in matching cards depicting bidimensional objects (e.g., red rabbits and blue boats) to one of two target cards. In a first block of trials, children are required to match cards according to one dimension (e.g., shape); In the second block (post-switch), they are required to sort stimuli according to the other dimension (here, color). Studies on this task in young children have shown that they succeed in sorting cards according to the first dimension, but fail to switch to the second rule after sorting by the first, and perseverate to match stimuli following the first rule. Marcovitch et al. (2007, 2010) tested the hypothesis that failure in the post-switch block was due to a flaw in maintenance of the goal, here of the matching rules, by manipulating the frequency of “conflict” cards in the post-switch block. Conflict cards require opposite matching depending on the rule to be used (because they match one target card on one dimension and the other on the other dimension). A high proportion of these cards thus lead to a greater need for goal maintenance, whereas a low proportion, involving many no-conflict cards that can be sorted independently of the rule to be applied, makes goal maintenance more demanding. As expected, preschoolers’ performance was worse when the frequency of conflict cards was low. Hence, despite understanding task instructions, young participants may fail to execute them effectively, a phenomenon that is referred to as “goal neglect” (Duncan et al., 1996). Chevalier and Blaye (2009) investigated the critical role of the activation of a task goal representation by manipulating task cues in a task-switching paradigm requiring participants to switch between shape- and color-matching rules. The authors graded the transparency of task-cues (i.e., the degree of association between cues and goals) and found that arbitrary cues made it more difficult for 5- and 6-year-old children to activate a representation of what to do next. Interestingly, the effect of cue transparency decreased in older children and adults, thereby suggesting that preschoolers’ struggle to translate arbitrary cues into task goals might reflect lower flexibility in comparison to older children. The nature of the changes contributing to the development of both the activation and maintenance of goal representations remains to be explored.

Recent research has evidenced age-related qualitative changes in control strategies that might promote the development of cognitive flexibility (Chevalier et al., 2011, 2013) and working memory (Camos and Barrouillet, 2011) from preschool to school ages. For instance, Camos and Barrouillet (2011) observed changes from a strategy of passive maintenance of memoranda in preschoolers, to a strategy of refreshing in school-age children. Using a flexibility task, Chevalier et al. (2013) produced the first findings suggesting a difference between 5 year-olds and 10 year-olds in goal representation and maintenance strategies. In addition to the task cues that indicated which task to perform next, as in the traditional task-switching paradigm, they provided transition cues specifying the nature of the transition between two consecutive trials: task repetition vs. task alternation. These transition cues were helpful for the younger participants, but proved to be detrimental to 10-year-olds’ flexibility scores, thereby suggesting that the two age groups employed different strategies in task–cue processing, and hence in goal representation. In the present paper, we further explore the nature of the changes that underlie developmental improvements in children’s ability to activate and maintain goal representations. Although developed to account for adult control, the Dual Mechanisms of Control theory (DMC theory, Braver et al., 2001, 2007; Braver and Barch, 2002; Braver, 2012) offers a theoretical framework for examining this question. Specifically, this approach offers an account of the way individuals retrieve and maintain goal-related information, and use it to guide processing (Braver, 2012). The DMC theory makes a qualitative distinction between two modes of control engaged under conditions of interference. It is noteworthy that interference can be induced by either irrelevant stimulus information or irrelevant dominant responses. These two control modes, respectively, called “proactive” and “reactive” have different temporal dynamics and neural substrates. The use of a proactive mode of control involves not only the retrieval of a representation of the goal in advance of the stimuli requiring a response, but also the active maintenance of this representation in working memory in order to bias processing towards task-relevant information. In contrast, with a reactive form of control, the goal is retrieved “just in time,” after the occurrence of the stimulus and its representation is transiently maintained in working memory.

Empirically, the two forms of control are assessed using the AX-Continuous Performance Task (AX-CPT, Braver et al., 2001). In this paradigm, cue–probe pairs are presented sequentially. Participants have to give a target vs. non-target response to each probe stimulus based on the cue stimulus presented immediately before it. In adults and older children, letters are used as cues and probes. At probe onset, participants are required to press one of two response keys, associated to either target or non-target responses. A target response is required when an A cue is followed by an X probe (AX target trials, thus the name AX-CPT), whereas non-target responses are to be given for all other cue–probe pairs (AY, BX, and BY trials, where Y and B represent any letters other than A and X). AX trials make up 70% of trials, while the frequency of each of the other three types of non-target trials is 10. Formally, since both AY and BX trials involve one letter that is strongly associated with the target response whereas a non-target response is expected, they could be considered as both requiring inhibition and then, could lead to similar performance (e.g., Paxton et al., 2008). The point of analyzing AX-CPT performance, however, is to reveal the pattern of differences between these two trial types. This pattern is considered as an index of the degree to which participants’ attention is drawn to the cue. Participants who use a proactive form of control engage in active preparation of their response to the probe when they see the cue. Hence, as the high proportion of AX trials creates a strong expectancy to give a target response it is detrimental to performance when the A cue appears and it is not followed by an X probe (i.e., AY trials). Indeed, this situation is specifically costly in terms of inhibition because participants have to reject the tendency to give a target response to the Y probe. The high AX trials’ frequency also induces a bias to produce a target response when an X probe is not preceded by an A cue (i.e., BX trials). Therefore, responding correctly to BX trials requires participants to actively maintain the B cue: because orienting attention towards B cue through active maintenance has the effect of inhibiting goal-irrelevant information, it aids participants to reject the strong tendency to give a target response to the X probe. The reverse pattern is expected in participants who have difficulty using goal-related information (i.e., who exercise reactive control): they do not anticipate their response to the probe according to the cue and make their decision only after the probe display. Because participants using reactive control do not actively maintain the cue during the cue–probe delay, they do not need to overcome the strong bias that an A cue is followed by an X probe. Hence, the use of reactive control should lead to higher performance on AY trials. By contrast, in order to produce a correct non-target response to X probes which follow an invalid cue (i.e., BX trials), participants have to retrieve the cue that they did not actively maintain in order to inhibit their tendency to give a target response when seeing the X probe. In sum, proactive control is typically evidenced by better performance on BX trials than on AY trials, while reactive control is reflected by better performance on AY than BX trials. It should be noted that performance on BY trials is not expected to differ between proactive and reactive participants, as neither the cue nor the probe is associated to a target response on this kind of trial.

Data from studies in adults investigating the relations between mode of control and working-memory on the one hand, and form of control and neural substrates on the other hand, suggest converging predictions on what could be the development of the dynamics of control. There is empirical evidence that working memory capacity plays a role in the temporal dynamics of control in adults. For instance, Redick (2014) showed in young adults that individuals with high working memory capacity tend to use a proactive form of control more often than individuals with low working memory capacity. The increase in working memory capacity over childhood (Gathercole et al., 2004) suggests one reason why younger children should encounter more difficulties using proactive control than older ones. Moreover, according to the DMC theory, proactive control is subserved by a phasic signal from the dopaminergic (DA) system prior to stimulus onset and by sustained activation of the lateral prefrontal cortex (PFC), a region that is known to be involved in the active maintenance of goal-related information. By contrast, reactive control does not involve a burst of DA activity, but instead involves transient activation of the lateral PFC when triggered by critical stimuli. In this case, the reactivation of goal-related information requires either the detection of interference through additional conflict monitoring regions such as the anterior cingular cortex (ACC), or the retrieval of associations through temporal or cortical brain areas. Given that the frontal lobes are known to be the last brain regions to develop, reaching maturity only in adolescence (Casey et al., 2000), researchers have hypothesized that younger children’s less efficient executive control might be related to the lesser powers of proactive control resulting from the immaturity of their frontal cortex (Braver, 2012; Munakata et al., 2012).

It is noteworthy that the developmental course of working memory and neural deterioration in aging suggests symmetrical developmental predictions. These predictions have received some empirical support: a shift from a proactive to a reactive mode of control with aging has been observed using both behavioral and neurophysiological measures (Braver et al., 2001, 2005; Paxton et al., 2008). The dynamics of control during childhood, by contrast, remain under-investigated, with only two studies addressing this question in children older than 8 (Lorsbach and Reimer, 2008, 2010) and only one contrasting 3.5- and 8-year-olds (Chatham et al., 2009). Lorsbach and Reimer (2010) found that children between the ages of 9 and 11 already engaged a proactive form of control. They observed a developmental increase in efficiency of this form of control only for a long cue–probe interval, suggesting that goal maintenance mechanisms are involved in the development of executive control during childhood. Chatham et al. (2009) drew similar conclusions in younger children. The authors used an adapted version of the AX-CPT paradigm with pictures instead of letters. Pupillometry measures and behavioral observations both revealed that 8-year-olds children engaged in intense mental efforts during the cue–probe interval, thereby suggesting that they struggled to actively maintain the cue in working memory. Younger children (3.5 years old) did not show any maintenance-related effort during this interval, but instead showed a reactive peak during probe display on BX trials. Although these data suggest a shift from reactive to proactive control during childhood, the turning point of these qualitative changes is unclear due to the large age gap (i.e., 3.5- vs. 8-year-olds) between the two groups. Moreover, the task used differed from the standard AX-CPT task in ways that might affect interpretations of the patterns of behavior. Not only did the task involve only two cues and two probes instead of the great diversity of letters referred to as B and Y in the standard AX-CPT, but also, in contrast to the arbitrariness of the cue–probe associations in the standard task, here it was contextualized in a story (e.g., As Spongebob (A) likes watermelon (X), a press on happy face is expected when Spongebob appears followed by the watermelon). In sum, it is unclear whether performances on this task are directly comparable to those obtained with the standard AX-CPT. Hence, further data using a task closer to the standard one is then required to enable a comparison between performance in young children and data previously obtained on older ones. In investigating a narrower age range, we expected to pinpoint the qualitative shift form reactive to proactive control.

In light of the finding of a substantial improvement in the ability to retrieve and maintain goal-related information in working memory between the ages of 5 and 6 years (Chevalier and Blaye, 2009; Chevalier et al., 2010; Camos and Barrouillet, 2011), we selected this age range to explore a potential shift from reactive to proactive control in children. Investigating this age range seems also particularly relevant with respect to Blackwell and Munakata’s (2013) interpretation of performance of 6-year-old children in a flexibility task, as revealing a shift from a reactive to a proactive mode of control. Moreover, since it has been suggested that working memory is critical in determining mode of control (Redick, 2014), we assessed these developmental differences in two different working memory load conditions by varying the length of the cue–probe delay. We used a new child-specific version of the AX-CPT, designed to be as similar as possible to the adult version of the task. Because this study is the first to investigate the dynamics of control in the age range of 5–6 years, alternative predictions can be made. First, there could be a shift from reactive to proactive control, which would then be evidenced by the typical pattern of reactive control in the younger group, with better performance on AY trials than on BX trials, and the reverse pattern in the older, proactive group. Second, it is also plausible that both age groups already use proactive control: in this case, changes between the ages of 5 and 6 would be evidenced by greater efficiency in retrieving and actively maintaining goal-related information. This should be reflected by increased difficulties in inhibiting a target response to the Y probe when presented following the A cue, and/or better ability to anticipate the need for a non-target response when presented along with a B cue: Third, both age groups could perform the task using a reactive mode of control. In this case, children may have difficulty anticipating the need for a non-target response when B cue is presented, but perform better when an A cue is followed by a non-target Y probe. If two profiles (proactive vs. reactive) would be observed, we hypothesized that children using a proactive control should demonstrate higher speed of processing, especially in the more demanding situation. This assumption was based on research on working memory that showed that participants associated with greater memory span (i.e., those better able to maintain information) are the faster ones (e.g. Barrouillet et al., 2009).

Following Lorsbach and Reimer’s (2010) observations in older children, we expected the differences between the two age groups to increase under conditions of high working memory load (long cue–probe delay). Finally, in order to track quantitative changes, we used an index of context sensitivity (*d*′) susceptible to provide a more graded picture of the extent to which children make target response to the X probe according to the cue presented ahead. One may hypothesize that sensitivity to cue information increases from the age of 5 to 6. As sensitivity to cue information can rely on proactive maintenance or reactive retrieval of the cue to guide response to X probe, we hypothesize a reduction of this sensitivity when the cue–probe delay increases because the high working-memory load in this case may hinder cue maintenance.

## MATERIALS AND METHODS

### PARTICIPANTS

Twenty-nine 5-year-olds (*M* = 5.80, SD = 0.26; 60% female) and twenty-eight 6-year-olds (*M* = 6.70, SD = 0.24; 56% female) were recruited from two French preschools and two French primary schools. Parental consent was given for all children, and the experiment was administered individually in a quiet room at the school. Most children were Caucasian and came from middle-class backgrounds, although no data were collected on race and socioeconomic status. Two additional preschoolers and one first-grader also began the experiment but were excluded from analyses because they were disturbed by an unexpected event in the room or they decided to stop the task while in progress.

### MATERIALS AND PROCEDURE

We created a child-adapted AX-CPT, replacing the letter stimuli from the original task with black-and-white drawings of animals. The animals were chosen on the basis of identification and naming norms established in 5-year-olds (Chalard et al., 2003; Cannard et al., 2005). As children performed the task twice for each delay condition, two different sets of 13 black-and-white drawings of animals were used in order to prevent boredom. The use of the two sets was counterbalanced across the two conditions. As for letters used in the classic version of the AX-CPT, the animals used for target trials in each set (A cues and X probes) were randomly chosen among each animal list and maintained constant for all participants^1^.

Before performing the task, we made sure that all the participants could name each of the animals used as stimuli. AY and BX non-target trials consisted in 12 possible combinations of animal pairs, and BY non-target trials consisted in 132 possible combinations of animal pairs. Task instructions were provided to children as follows: “You will see animals on the screen; these animals run in pairs, one after the other (“ces animaux courent deux par deux, l’un après l’autre”).” In one set of animals, children were given the following instruction: “when you first see the hen (A cue) and then the cat (X probe), press the green button, otherwise press the red one.” For the other set of animals, they were told “when you first see the frog (A cue) and then the donkey (X probe), press the green button, otherwise press the red one.” Children were instructed to respond as quickly and accurately as possible. To ensure that they had memorized the instructions, they were twice shown 4 pairs of sheets of paper mimicking four successive displays of cue and probe combinations on the screen (i.e., AX, AY, BX, BY), once before moving on to the computer training, and once at the end of each session. For each pair, children were questioned about the correct response button to press and were asked to justify their answer to test whether they remembered the rule. All children succeeded in recalling the instructions (showing the correct response button and justifying their response by recounting the rules).

**FIGURE 1:**
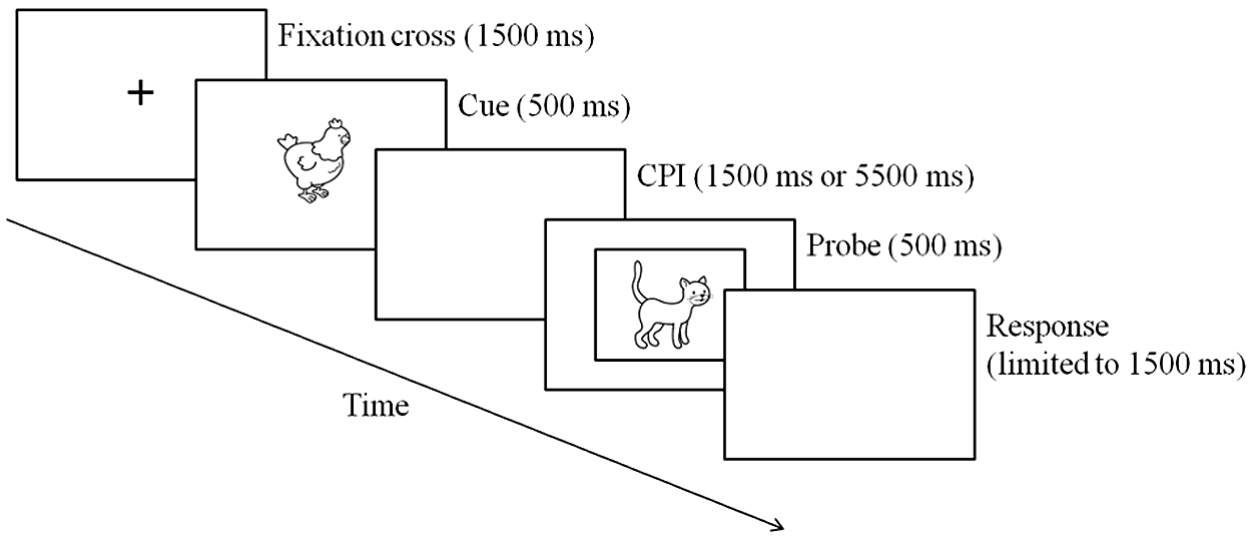
**Example of an AX trial sequence**.

Children were tested individually in two cue–probe delay conditions (1500 ms for the short delay vs. 5500 ms for the long delay) in a counterbalanced order across participants, distributed into two sessions lasting approximately 20–30 min each. A 30-min break was given between the two conditions, during which participants returned to their classroom. Pictures were presented sequentially on a HP Compaq 9000 laptop, using the E-Prime software (Psychology Software Tools, Inc., 2007). Each trial began with the presentation of a fixation cross on the screen for 1500 ms. A cue was then presented at the center of the screen for 500 ms (the first animal, A or B, in the cue–probe pair), followed by a blank screen displayed according to the cue–probe delay (short or long). After this delay, a probe appeared at the center of the screen for 500 ms (the second animal, X or Y, in the cue–probe pair; see **Figure 1**). All probes were framed by a fine black line in order to help children differentiate between cues and probes and decide unambiguously when a response was expected. To encourage children to respond quickly, a warning tone was played when responses exceeded a 1500 ms time limit. Seventy percent of trial were AX target trials, and each of the three kinds of non-target trials (AY, BX, and BY) each made up 10% of trials. The pairs of pictures were presented pseudo-randomly; the number of AX trials in a row never exceeded four. Each delay condition involved a training phase followed by an experimental phase. The training phase included three blocks of 20 trials (14 AX trials, two AY, two BX, and two BY) and the testing phase included four blocks of 30 trials (21 AX trials, three AY, three BX, and three BY), yielding a total of 180 trials.

## RESULTS

The main effects of condition order and animal set were not significant, and these variables did not interact significantly with other variables of interest (all *p* > 0.10): they thus were not included in further analyses. Following Lorsbach and Reimer (2008, 2010), we computed different sets of analyses on target trials (AX) and non-target trials (AY, BX, and BY), because they do not involve the same number of trials. The RT on each correct trial was then standardized by subtracting the participant’s overall mean to each correct RT and dividing the difference by the same participant’s SD. Mean *z*-scores were then calculated for each participant in each condition: negative *z*-scores reveal fast RTs whereas positive *z*-scores correspond to slow RTs. This standardization corrects for individual differences in speed of processing. For clarity, **Table 1** presents a summary of error rates, correct response times and mean *z*-scores. Importantly, because the reliability of error rates is often higher than that of RTs in preschoolers (e.g., Chevalier and Blaye, 2009), analyses on error rates are reported first.

**Table 1:** Mean error rates, correct RTs and *z*-scores by age group and trial type.

	Trial condition							
	Short cue-probe delay condition (1500 ms)				Long cue-probe delay condition (5500 ms)			
*Age group*	*AX*	*AY*	*BX*	*BY*	*AX*	*AY*	*BX*	*BY*
**5-year-olds**
% E	5.6 (3.9)	24.9 (19)	21.8 (20)	13.4 (16)	9.3 (9.4)	37.3 (25)	30.1 (27)	12 (15)
RT	825 (142)	1006 (128)	862 (204)	801 (177)	914 (146)	1098 (190)	818 (203)	902 (202)
Mean *z*-score	-0.18 (0.24)	+0.47 (0.35)	-0.02 (0.46)	-0.27 (0.31)	+0.11 (0.23)	+0.79 (0.38)	-0.16 (0.55)	+0.07 (0.47)
**6-year-olds**
% E	3.3 (2.7)	29 (29.8)	6.7 (7.2)	4.2 (7.4)	6.3 (4.4)	36.5 (33.6)	12.8 (11.9)	5.2 (8.2)
RT	687 (154)	893 (153)	670 (227)	663 (183)	762 (154)	984 (89)	693 (207)	710 (178)
Mean *z*-score	-0.17 (0.23)	+0.83 (0.87)	-0.25 (0.56)	-0.28 (0.47)	+0.14 (0.22)	+0.97 (0.43)	-0.18 (0.60)	-0.10 (0.47)

SDs are in parentheses.

### AX TARGET TRIALS

Two similar analyses of variance were run on error rates and mean *z*-scores, with age group (5-year-olds vs. 6-year-olds) as a between-subjects variable and delay (1500 ms short vs. 5500 ms long) as a within-subjects variable.

Age was found to have a significant main effect on error rates, *F*(1,55) = 4.66, *p* < 0.05, $\eta_{p}^{2}$ = 0.07, indicating more errors in 5-year-olds (*M* = 7.4%) than in 6-year-olds (*M* = 4.8%), but not on *z*-scores, *F*(1,55) = 1.74, *p* = 0.18. The results also revealed a main effect of delay, both on error rates, *F*(1,55) = 13.01, *p* < 0.001, $\eta_{p}^{2}$ = 0.19, with higher error rates at the long delay (*M* = 7.8%) than at the short one (*M* = 4.4%), and on *z*-scores, *F*(1,55) = 227.72, *p* < 0.001, $\eta_{p}^{2}$ = 0.80, indicating faster response times for the short delay (*M* = -0.17) than for the long delay (*M* = 0.88 ms). However, the Age × Delay interaction was not significant, either for error rates or for *z*-scores, *F*(1,55) = 0.12, *p* = 0.72, and *F*(1,55) = 1.48, *p* = 0.22, respectively.

To summarize, error rates on AX trials significantly decreased with age, while latencies on correct trials remained stable between the two age groups. Furthermore, longer delays had a detrimental effect on accuracy and latencies on AX trials.

### AY, BX, AND BY NON-TARGET TRIALS

Two analyses of variance were run, following the same design for both error rates and *z*-scores. They involved age group (5-year-olds vs. 6-year-olds) as a between-subjects variable and delay (1500 ms, short vs. 5500 ms, long) and trial type (three types: AY, BX, BY) as within-subjects variables. Because two 5-year-olds and four 6-year-olds produced wrong responses to all trials of one type (i.e., all AY or all BX trials) in the long delay condition, their *z*-score for this type of trial was replaced by the mean *z*-score for their age group to increase statistical power.

A main effect of age was observed on error rates only, *F*(1,55) = 5.07, *p* < 0.05, $\eta_{p}^{2}$ = 0.08, revealing that 5-year-olds committed more errors (*M* = 23.2%) than 6-year-olds (*M* = 15.7%).Trial type had a significant effect on both performance measures, *F*(2,110) = 33.22, *p* < 0.001, $\eta_{p}^{2}$ = 0.37, for error rates and *F*(2,110) = 121.46, *p* < 0.001, $\eta_{p}^{2}$ = 0.68, for *z*-scores. Children committed more AY errors (*M* = 31.9%) than BX errors (*M* = 17.8%) thereby revealing their use of a proactive mode of control. In addition, BY trials (*M* = 8.7%) led to fewer errors than AY, *F*(1,55) = 55.41, *p* < 0.001, $\eta_{p}^{2}$ = 0.50, and BX trials, *F*(1,55) = 33.58, *p* < 0.001, $\eta_{p}^{2}$ = 0.37. Turning to *z*-scores, planned comparisons indicated that latencies were longer on AY trials (*M* = 0.77) than on BX (*M* = -0.15), *F*(1,55) = 120.08, *p* < 0.001, $\eta_{p}^{2}$ = 0.68, and BY trials (*M* = -0.14), *F*(1,55) = 211.34, *p* < 0.001, $\eta_{p}^{2}$ = 0.79, whereas the latter two did not differ, *F*(1,55) = 0.04, *p* = 0.84. Analyses of response time patterns thus confirmed the above conclusion on error rates. The results also revealed a main effect of delay on error rates, *F*(1,55) = 8.59, *p* < 0.01, $\eta_{p}^{2}$ = 0.13, revealing higher error rates at the long delay (*M* = 22.3%) compared to the short delay (*M* = 16.6%). A main effect of delay on *z*-scores was also observed, *F*(1,55) = 4.14, *p* < 0.05, $\eta_{p}^{2}$ = 0.07, with shorter latencies at the short delay (*M* = 0.07) than the long delay (*M* = 0.23).

Turning to interactions for both measures, only two interactions revealed significant. The interaction between age and trial type was significant on error rates, *F*(2,110) = 4.86, *p* < 0.01, $\eta_{p}^{2}$ = 0.08, and on *z*-scores, *F*(2,110) = 5.15, *p* < 0.01, $\eta_{p}^{2}$ = 0.08. A Delay × Trial Type interaction was obtained both on error rates and on *z*-scores, *F*(2,110) = 4.67, *p* < 0.05, $\eta_{p}^{2}$ = 0.07, and *F*(2,110) = 4.67, *p* < 0.05, $\eta_{p}^{2}$ = 0.07. They are explored further below.

#### Does age affect the temporal dynamics of control?

**FIGURE 2:**
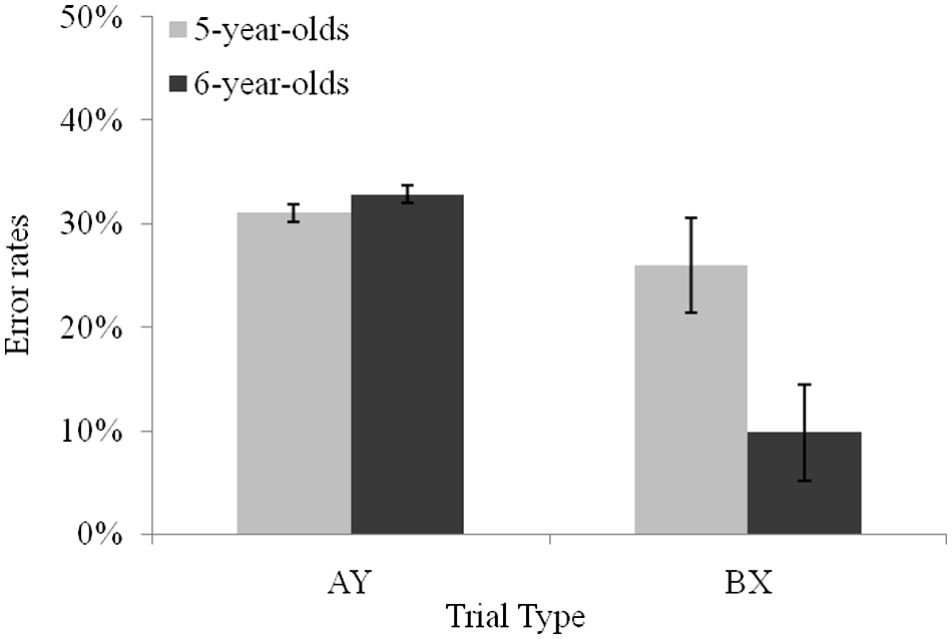
**Performance of 5- and 6-year-olds on AY and BX non-target trial types.** Error bars reflect SEs of the means.

Planned comparisons revealed that younger children produced more errors than older children on BX trials (*M* = 25.9%, and *M* = 9.8%, respectively), *F*(1,55) = 18.10, *p* < 0.001, $\eta_{p}^{2}$ = 0.24, and on BY trials (*M* = 12.6%, and *M* = 4.7%, respectively), *F*(1,55) = 7.41, *p* < 0.01, $\eta_{p}^{2}$ = 0.11, whereas error rates between the two age groups did not differ on AY trials, *F*(1,55) = 0.07, *p* = 0.79. Planned comparisons in each age group indicated that the typical proactive pattern observed when considering all participants was observed in the older group only with more errors on AY (*M* = 32.8%) than on BX trials (*M* = 9.8%), *F*(1,55) = 20.85, *p* < 0.001, $\eta_{p}^{2}$ = 0.27 (see **Figure 2**). In contrast, no significant difference was observed between AY and BX trials (*M* = 31% and *M* = 25.9%, respectively) in 5-year-olds, *F*(1,55) = 1.06, *p* = 0.30. Turning to *z*-scores, planned comparisons revealed that both 5- and 6-year-olds presented longer latencies on AY than on BX trials, *F*(1,55) = 37.97, *p* < 0.001, $\eta_{p}^{2}$ = 0.40, and *F*(1,55) = 86.64, *p* < 0.001, $\eta_{p}^{2}$ = 0.61, respectively. However, the difference between latencies on AY and BX trials increased from age 5 to 6, *F*(1,55) = 5.38, *p* < 0.05, $\eta_{p}^{2}$ = 0.08. The larger difference between AY and BX trials performance was due a difference between age groups latencies on AY trials: on this trial type, 6-year-olds produced slower latencies (*M* = 0.90) than 5-year-olds (*M* = 0.63), *F*(1,55) = 6.89, *p* < 0.05, $\eta_{p}^{2}$ = 0.11. Latencies on BX trials (*M* = -0.09 and *M* = -0.21, respectively) and BY trials (*M* = -0.10 and *M* = -0.19, respectively) did not differ between the younger and the older age group, *F*(1,55) = 1.23, *p* = 0.27, and *F*(1,55) = 1.80, *p* = 0.18, respectively.

Considering that the lack of difference between performance on AY and BX trials in the 5-year-old group could not be interpreted, we explored their performance on these trials further in order to investigate whether there might be two subgroups with differing modes of control. We performed a median split based on the critical difference between the error rates observed in these two kinds of trials. It was plausible that none of the subgroups used a reactive mode of control, and that the average difference between AY and BX trials error rates would remain close to zero in both subgroups. Alternatively, the subgroups could differ in their mode of control: one could have performed the task using reactive control, in which case their AY-BX average should be significantly negative, while the other used a proactive mode and thus should have a significantly positive AY-BX average. An ANOVA was run with group (above vs. below the median difference score) as a between-subjects factor and trial type (AY, BX) and delay (1500 ms short vs. 5500 ms long) as within-subjects factors.

**FIGURE 3:**
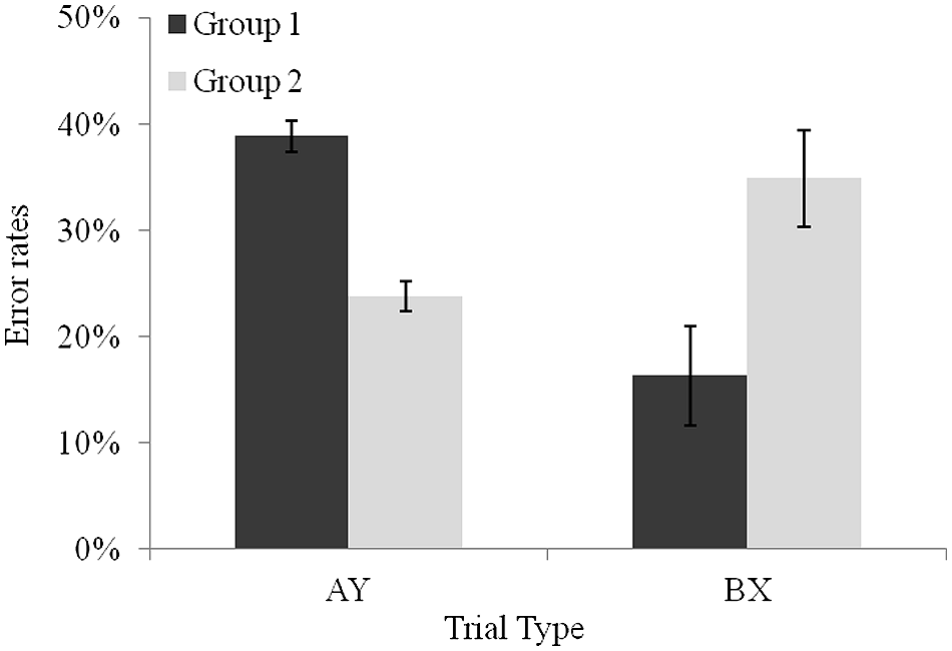
**Performance of the two groups of 5-year-olds on AY and BX non-target trial types.** Error bars reflect SEs of the means.

The analysis revealed a significant interaction between trial type and group, *F*(1,27) = 32.29, *p* < 0.001, $\eta_{p}^{2}$ = 0.54. Planned comparisons indicated that on average, participants in the group above the median made more errors on AY trials (*M* = 38.8%) than on BX trials (*M* = 16.3%), *F*(1,27) = 27.97, *p* < 0.001, $\eta_{p}^{2}$ = 0.50, which suggests a proactive use of control. The reverse pattern was observed in the group below the median, *F*(1,27) = 7.31, *p* < 0.05, $\eta_{p}^{2}$ = 0.21, with more errors in BX trials (*M* = 34.9%) than of AY trials (*M* = 23.7%), thereby revealing the use of a reactive form of control (see **Figure 3**)^2^. We also tested whether this contrast between the two subgroups would persist when considering *z*-scores. A new ANOVA was run with group (above vs. below the median difference score) as a between-subjects factor and trial type (AY, BX) and delay (1500 ms short vs. 5500 ms long) as a within-subjects factor. A significant interaction between trial type and group was obtained, *F*(1,27) = 5.11, *p* < 0.05, $\eta_{p}^{2}$ = 0.15. Both groups were slower on AY trials than on BX trials, *F*(1,27) = 53.82, *p* < 0.001, $\eta_{p}^{2}$ = 0.66, and *F*(1,27) = 18.82, *p* < 0.001, $\eta_{p}^{2}$ = 0.41. However, planned comparisons revealed that the difference between latencies on AY and BX trials was larger in the above-median-group than in below-median group, *F*(1,27) = 5.11, *p* < 0.05, $\eta_{p}^{2}$ = 0.15. Moreover, in order to gain a better understanding of children’s proactive vs. reactive characteristics; we compared children’s speed of processing of the two subgroups through latencies on BY trials. This trial is considered as a baseline condition because both cue and probe are associated to non-target responses. Children shown to use reactive control were marginally slower in the more demanding condition (i.e., in the long delay) than children engaging proactive control (*M* = 0.22, and *M* = -0.08, respectively), *t*(27) = -1.84, *p* = 0.06.

In summary, age-related differences were found both on error rates and on *z*-scores. Error rates analyses revealed important inter-individual differences within the 5-year-olds group and altogether these findings shaped a developmental path towards an increasing efficiency of proactive control with age.

#### Does the cue maintenance delay affect the temporal dynamics of control?

Planned comparisons revealed more errors with the long delay than with the short one on AY trials (*M* = 36.9% and *M* = 26.9%, respectively), *F*(1,55) = 9.76, *p* < 0.01, $\eta_{p}^{2}$ = 0.15, and on BX trials (*M* = 21.5% and *M* = 14.2%, respectively), *F*(1,55) = 5.28, *p* < 0.05, $\eta_{p}^{2}$ = 0.08, whereas error rates on BY did not differ between the two delays (*M* = 8.6% and *M* = 8.8%, respectively), *F*(1,55) = 0.01, *p* = 0.90. Turning to *z*-scores, planned comparisons showed longer latencies on AY trials with a long delay than with a short delay (*M* = 0.88 and *M* = 0.65, respectively), *F*(1,55) = 5.04, *p* < 0.05, $\eta_{p}^{2}$ = 0.08, whereas *z*-scores on BX trials did not differ between the two delay conditions (*M* = -0.17 and *M* = -0.14, respectively), *F*(1,55) = 0.12, *p* = 0.72.

### THE DEVELOPMENT OF CONTEXT SENSITIVITY (d′ SCORES)

In order to assess the development of children’s sensitivity to the preceding context when presented with an X probe, the signal detection index *d*′ was computed (Lorsbach and Reimer, 2008, 2010) corresponding to a ratio between the proportion of correct responses on AX trials (hits) and the proportion of incorrect target responses on BX trials (false alarms). It should be noted that this index does not indicate whether participants use reactive or proactive control to perform the task since false alarms on BX trials can be either due to failures in actively maintaining the B cue, or by a failure to retrieve B cue after the occurrence of X probe. The higher the value of *d*′, the more efficiently the participant used previous goal-related information (A or non-A) to produce a target or a non-target response in response to the X probe. To compare whether 5-year-old children differed from 6-year-olds in their sensitivity to cue information, we ran an ANOVA on *d*′ values with age (5-year-olds vs. 6-year-olds) as a between-subjects variable and delay (1500 ms, short vs. 5500 ms, long) as a within-subjects variable. A main effect of age was observed, *F*(1,55) = 23.86, *p* < 0.001, $\eta_{p}^{2}$ = 0.18, with larger *d*′ scores in 6-year-olds than in 5-year-olds (*M* = 0.39 and *M* = 0.31, respectively). A main effect of delay was also found, *F*(1,55) = 12.89, *p* < 0.001, $\eta_{p}^{2}$ = 0.18, showing larger *d*′ scores in the short than in the long delay condition (*M* = 0.37 and *M* = 0.33, respectively). However, the interaction between these two variables was not significant, *F*(1,55) = 0.11, *p* = 0.73.

Altogether, results on *d*′ scores revealed an increase in children’s sensitivity to cue information between the ages of 5 and 6. In addition, all age groups showed reduced sensitivity to cue information under the long cue–probe delay condition.

## DISCUSSION

It is now well established that executive control dramatically develops before the age of 6. Several recent studies converge to suggest that this progress might be sustained by a growing efficiency in activating one’s task goal and in maintaining its representation to guide the production of a response. However, the extent to which these changes are supported by a shift in the mode of control used remains under-investigated. The current study aimed to (a) explore the temporal dynamics of executive control at the ages of 5 and 6; and (b) study whether manipulating the working memory load influences these dynamics or modulates their efficiency. Our results provide empirical evidence for both qualitative and quantitative changes in the dynamics of control. Importantly, the findings reveal a qualitative shift from reactive to proactive control at the age of 5, as well as graded changes in proactive control from 5- to 6-year-olds. With respect to our second aim, increasing the working memory load did not prevent the active maintenance of goal information; however, it reduced children’s sensitivity to the nature of the cue presented earlier. The present results are in accordance with those of previous studies attesting to developmental improvements in activation and maintenance of goals during childhood (Marcovitch et al., 2007, 2010; Chatham et al., 2009; Chevalier and Blaye, 2009; Lorsbach and Reimer, 2010). Further, our findings reveal that the improvement between the ages of 5 and 6 reflects both qualitative and quantitative changes in control. Together, the two groups of children demonstrated the engagement of proactive control, both on error rates and latencies when contrasting their performance on BX and AY trials. We recall that proactive control is reflected by worse performance on AY trials since maintaining cue information is detrimental in this condition due to the high frequency of AX pairs in the task that induces a strong expectation of a target response which then needs to be inhibited when the Y probe is displayed. Whereas this pattern was maintained when considering the older group of children, the picture was less clear-cut in 5-year-olds, who produced similar performance on both types of trials. Further analyses, discussed below, revealed that this mixed picture was probably the consequence of inter-individual differences among this age group. More gradual, quantitative differences were observed between younger and older children. As expected, 6-year-olds appeared more sensitive to cue information in deciding whether or not to produce a target response, corresponding to an increased sensitivity index and less errors on BX trials. They also took longer than 5-year-olds in selecting the non-target response on AY trials. Altogether, these results suggest that context information was better maintained and guided more closely responses in 6-year-olds.

According to the DMC theory, the activation and maintenance of goal representation is underlain by neurobiological mechanisms (lateral PFC and DA system). Proactive control involves a sustained activation of the lateral PFC through a phasic signal of DA, which regulates access of information to enable the active maintenance of task-relevant goal information. In contrast, reactive control is related to a transient activation of PFC because bursts of DA do not occur. During the last decades, behavioral and anatomical studies provided evidence that the PFC and DA system reach maturity during adolescence (Casey et al., 2000; Posner et al., 2012) but dramatically develop during early childhood (Giedd et al., 1999; Rueda et al., 2004, 2005; Moriguchi and Hiraki, 2011). How can children, from at least the age of 6, already use proactive control? It can be argued that the neural substrates underlying proactive control in young children might, at least partially, differ from those activated in adolescents and adults due to their still immature PFC and DA system and/or overall neural activation could be larger than in adults. Alternatively, as suggested by the quantitative indexes of an increase in proactive control efficiency between the ages of 5 and 6, it seems plausible that this form of control is still far from optimal in the older group and hence could be sustained by a still partially immature PFC. Neurophysiological evidence in the field of the development of executive control bear support to each of these hypotheses (see Banich et al., 2013; and Larson et al., 2012; for data compatible with the first and second hypothesis, respectively). Further studies are thus required to investigate the extent to which proactive control in children is subserved by neural substrates similar to adults’ proactive control.

A deeper investigation within the 5-year-old group revealed contrasting patterns on error rates with some children already engaging a proactive mode of control to perform the task, and others using a reactive mode. While bearing in mind the limitations of the approach used to set-up the subgroups – which may have reinforced inter-individual differences between the modes of control – this finding suggests that the age of 5 might correspond to a transition in the development of control, at least in situations involving an active maintenance and/or a retrieval of context information. In line with children studies arguing accuracy to be a more sensitive measure than RT (Diamond and Kirkham, 2005; Chevalier and Blaye, 2009), analyses on latencies failed to reveal distinct control modes in the two subgroups. However, these analyses evidenced graded differences in the efficiency of proactive control between the two 5-year-old subgroups that were in a direction consistent with findings on error rates. Although both subgroups took longer to correctly respond to AY than BX trials, this difference was more pronounced – as expected from more efficient users of a proactive mode of control – in the subgroup identified as proactive on the basis of error rates. As proactive control requires maintenance of information during the cue probe delay, while reactive control does not, we considered that reactive patterns could be produced by children less efficient at maintaining information. Research on the development of working memory has established correlations between working memory and speed of processing scores (e.g., Barrouillet et al., 2009; Camos and Barrouillet, 2011). Indeed, the two subgroups contrasted here revealed marginal differences in terms of speed of processing. As expected, children shown to use reactive control were slower in the more demanding condition (i.e., long delay). Although further investigation of their working memory capacities would be necessary, this finding offers a convergent pattern with the error rate analysis. We will discuss further the relations between mode of control and working memory when considering the effect of the delay between cue and probe. We now examine recent results published independently while this study was run that suggest that a shift between reactive and proactive control might occur one year later that is, at 6 years of age.

Blackwell and Munakata (2013) suggested that the dynamics of control can be evidenced by considering children’s performance in a three dimensional version of the DCCS (3-DCCS). In this task, participants have to sort tridimensional stimuli. This leads to three blocks of trials, each block corresponding to one type of sort imposed by the experimenter’s instructions (i.e., sorting first by shape, then by color, then by size). The authors’ reasoning is that children who succeed to switch from one block to another use a proactive control because they achieve to maintain the relevant sorting rule which is given only once at the beginning of each block in a highly interfering context due to the two other rules. By contrast, perseveration would reveal a difficulty of reactivating the correct sorting rule in this highly conflictive context, authorizing to consider perseverators as engaging a reactive control. It might be argued that the age difference in the transition from reactive to proactive control between this study and the current one is due to the index used: switching between tasks through post-switch accuracy vs. performance on AY and BX trials. However, on the one hand the AX-CPT is the most characteristic task to assess the dynamics of control, and on the other hand, Blackwell and Munakata’s (2013) findings revealed that the *a priori* categorization of switchers as proactive and perseverators as reactive was corroborated by their performance on a delayed matching task. Hence, a new question must be raised: could the differences between the two tasks used to contrast the two modes of control account for the one year difference to observe a shift across the two tasks? We contend that the 3-DCCS is more demanding in terms of active maintenance since the tridimensional stimuli trigger not only the currently relevant rules but also the two irrelevant ones. By contrast the AX-CPT makes proactive control easier to engage since participants do not encounter any stimuli during the cue-maintenance delay.

Given the limited working-memory capacity in young children, we hypothesized that increasing the working-memory load through lengthening the delay of cue maintenance would increase the working memory load and hence, would decrease children’s efficiency at using the cue to guide their response to the probe, thereby inducing a shift from a proactive to a reactive mode of control. The results did not support this hypothesis since no reversal of the pattern of control was observed. This could suggest that active maintenance of goal-related information from the cue is not the most critical determinant of the mode of control engaged, at least in the age groups studied. Instead, it could be more crucial to retrieve an explicit representation of the goal when seeing the cue. Recent research by Chevalier and Blaye (Chevalier and Blaye, 2009; Blaye and Chevalier, 2011) has pointed to the role of task–cue translation into goals in preschoolers’ performance on a flexibility task. By contrasting different types of task–cues that varied in their degree of transparency (i.e., the degree of association between cue and task goal), the authors demonstrated that preschoolers had specific difficulties to retrieve a representation of what they had to do next when arbitrary cues were used even though they were able to recall the meaning of the cues. Cues used in the AX-CPT are arbitrary; the pairs presented as target pairs (AX) or non-target pairs (AY, BX, and BY) are all arbitrarily composed and the expected response has no relation with the animals either (i.e., pressing a green or red button). Hence, it would be worth comparing the mode of control engaged in different versions of the AX-CPT by a same sample of preschoolers depending on whether the cues–probes–responses associations would arbitrary or meaningful. Such a meaningful version has been used by Chatham et al. (2009) but on different age groups. Interestingly, Lorsbach and Reimer (2010) interpreted 8-year-olds′ weaker proactive control, compared to older children, as arising from difficulties to transform the cue into a complete representation of the goal.

A more parsimonious interpretation of the lack of shift from one mode of control to another when contrasting the two cue–probe delays could be that, the two delays are either too much or not sufficiently demanding in terms of maintenance. The overall proactive control observed in the two age groups does not support the hypothesis of two delays that would be too demanding; however, this might be at least partly the case for the 5-year-old subgroup that was found to use a reactive mode of control in both delay conditions. Alternatively, one may assume that increasing the cue–probe delay without any additional information to process in the meanwhile is not sufficiently demanding to induce qualitative changes in control. It could be worth testing the effect of another form of WM load manipulation, namely varying the demand of a concurrent processing task during the cue-probe delay. Nevertheless, the absence of a shift in the dynamics of control when lengthening the cue probe delay does not mean a lack of impact of this manipulation. More graded measures revealed quantitative changes suggesting that manipulating the delay does affect the working memory load. Children’s efficiency in using the cue information to guide their response to the probe appeared to be lowered with longer delay. Hence, when goal-related information has to be actively maintained, preschool age children can encounter difficulties to use it, without demonstrating the use of a pure reactive mode of control.

In sum, the current study aimed to investigate some of the quantitative and qualitative changes in activation and maintenance of goal representation between 5 and 6 years of age that might sustain the development of executive control. Although two recent theoretical papers (Braver, 2012; Munakata et al., 2012) offered the hypothesis that the development of executive control could correspond to a shift from reactive to proactive control during childhood, empirical validation of this hypothesis remains scarce. The current study proposes a children-adapted version of the AX-CPT and suggests that such a shift might occur at 5 years of age. This new finding is somewhat at odds with the results obtained by Blackwell and Munakata (2013). These authors observed this transition one year later using a different task originally designed to assess flexibility. This décalage raises the question of the extent to which this reversal in the temporal dynamics of control depends on the task demand in terms of active maintenance of goal information. Future investigation of this question should lead to a more complex picture of the development of executive control than the probably too simplistic view suggesting that these two modes of control correspond to two developmental stages.

## AUTHOR CONTRIBUTIONS

Joanna Lucenet and Agnès Blaye designed the Experiment. Data collection was carried out by Joanna Lucenet. Joanna Lucenet drafted the manuscript and Agnès Blaye provided critical revisions. Joanna Lucenet and Agnès Blaye have all approved the final version of the manuscript and agree to be accountable for all aspects of the work.

## Conflict of Interest Statement

The authors declare that the research was conducted in the absence of any commercial or financial relationships that could be construed as a potential conflict of interest.

## Footnotes

1 One set of animals included a hen (A cue), a cat (X probe), a giraffe, a mouse, a crocodile, a horse, a cow, a sheep, a snake, a fish, a rabbit, a pig, and a lion. The other set of animals consisted in a donkey (A cue), a frog (X probe), a squirrel, a dolphin, a bee, a duck, a kangaroo, a rooster, a spider, a turtle, a monkey, a dog, and an elephant.

2 Although we acknowledge that the median-split approach may have maximized the chances of evidencing a positive difference in one subgroup vs. a negative difference in the other, such contrasted patterns of performance were not obtained when conducting the same method in 6-year-olds. The same analysis on error rates as the one run in 5-year-olds revealed a significant interaction between trial type and group, *F*(1,26) = 38.96, *p* < 0.001; $\eta_{p}^{2}$ = 0.59. More errors on AY than on BX trials were found in the group above the median, *F*(1,26) = 79.00, *p* < 0.001; $\eta_{p}^{2}$ = 0.75, whereas performance between AY and BX trials did not significantly differ in the group below the median (*p* = 0.95). This suggests that the median split in itself does not systematically lead to a conclusion of reactive control.
